# Implementation of core elements of antibiotic stewardship in long-term care facilities—National Healthcare Safety Network, 2019–2022

**DOI:** 10.1017/ash.2025.31

**Published:** 2025-03-24

**Authors:** Angelina Luciano, Sarah Kabbani, Melinda M. Neuhauser, Ti Tanissha McCray, Lindsay Robinson, Theresa Rowe, Katryna A. Gouin

**Affiliations:** 1 Chenega Corporation, on assignment to the Centers for Disease Control and Prevention, Atlanta, GA, USA; 2 Division of Healthcare Quality Promotion, National Center for Emerging and Zoonotic Infectious Diseases, US Centers for Disease Control and Prevention, Atlanta, GA, USA

## Abstract

In 2022, uptake of all seven Core Elements of Antibiotic Stewardship were reported by 83% of US long-term care facilities. Though 98% of facilities reported access to an electronic health record, less than one-third utilized it for tracking antibiotic use, suggesting opportunities to leverage electronic data for automated reporting.

## Introduction

In 2015, the Centers for Disease Control and Prevention (CDC) published the Core Elements of Antibiotic Stewardship for Nursing Homes, a framework for developing and implementing stewardship programs in long-term care facilities (LTCFs).^1^ In 2017, Centers for Medicare & Medicaid Services (CMS) regulatory requirements for implementing an antibiotic stewardship program went into effect.^2^ The uptake of the seven Core Elements in LTCFs increased following CMS regulatory requirements, from 43% uptake among the LTCFs submitting surveys to CDC’s National Healthcare Safety Network (NHSN) in 2016 to 71% in 2018.^3,4^ This report provides an update of the uptake of the Core Elements in LTCFs by facilities reporting to NHSN from 2019 to 2022. A description of facilities’ electronic health record (EHR) uptake and data sources used to track antimicrobial use (AU) in 2022 is also reported.

## Methods

We assessed the uptake of the CDC’s Core Elements of Antibiotic Stewardship among U.S. skilled nursing facilities and nursing homes that submitted a LTCF Component Annual Facility Survey to NHSN in 2019–2022. The survey is required among LTCFs reporting to the NHSN healthcare associated-infections modules^4^ and includes questions on facility characteristics, infection prevention and control practices, and antibiotic stewardship activities. It is completed each year by an authorized LTCF staff member, such as the infection preventionist or director of nursing.

The 10 survey questions related to antibiotic stewardship were mapped to the seven Core Elements (leadership, accountability, drug expertise, action, tracking, reporting, and education).^3,5^ “Uptake” of a Core Element was identified through an affirmative response to at least one corresponding survey question (https://arpsp.cdc.gov/about?tab=hospital-stewardship).

For the analysis, we excluded surveys that were missing responses to antibiotic stewardship survey questions and/or facility characteristics or if submitted from facilities in US territories. We described characteristics of facilities reporting in 2022: bed size, region, affiliation, ownership type, and EHR access. We described the reported uptake of all and individual Core Elements in each survey year. We conducted a sensitivity analysis to assess the uptake of the Core Elements among consecutive reporters, defined as LTCFs that submitted a survey in all four years of the study period (2019–2022), and compared the results to the primary analytic cohort of LTCFs enrolled at any time in 2019–2022. We also described the roles of the individual(s) responsible for the facility’s antibiotic stewardship program in 2022.

The 2022 NHSN LTCF Component Annual Facility Survey was modified to capture data sources used to track AU: pharmacy services, EHR, manual reporting, and other. We conducted a qualitative content analysis of the free-text output for facilities reporting “other” data sources to track AU. We reported the most common AU tracking systems across LTCFs in 2022. All quantitative analyses were conducted using SAS version 9.4 software (SAS Institute, Cary, North Carolina). This activity was reviewed by the CDC and was conducted consistent with applicable federal law and CDC policy.

^§§^45 C.F.R. part 46, 21 C.F.R. part 56; 42 U.S.C. Sect. 241(d); 5 U.S.C. Sect. 552a; 44 U.S.C. Sect. 3501 et seq.

## Results

**Figure 1.**
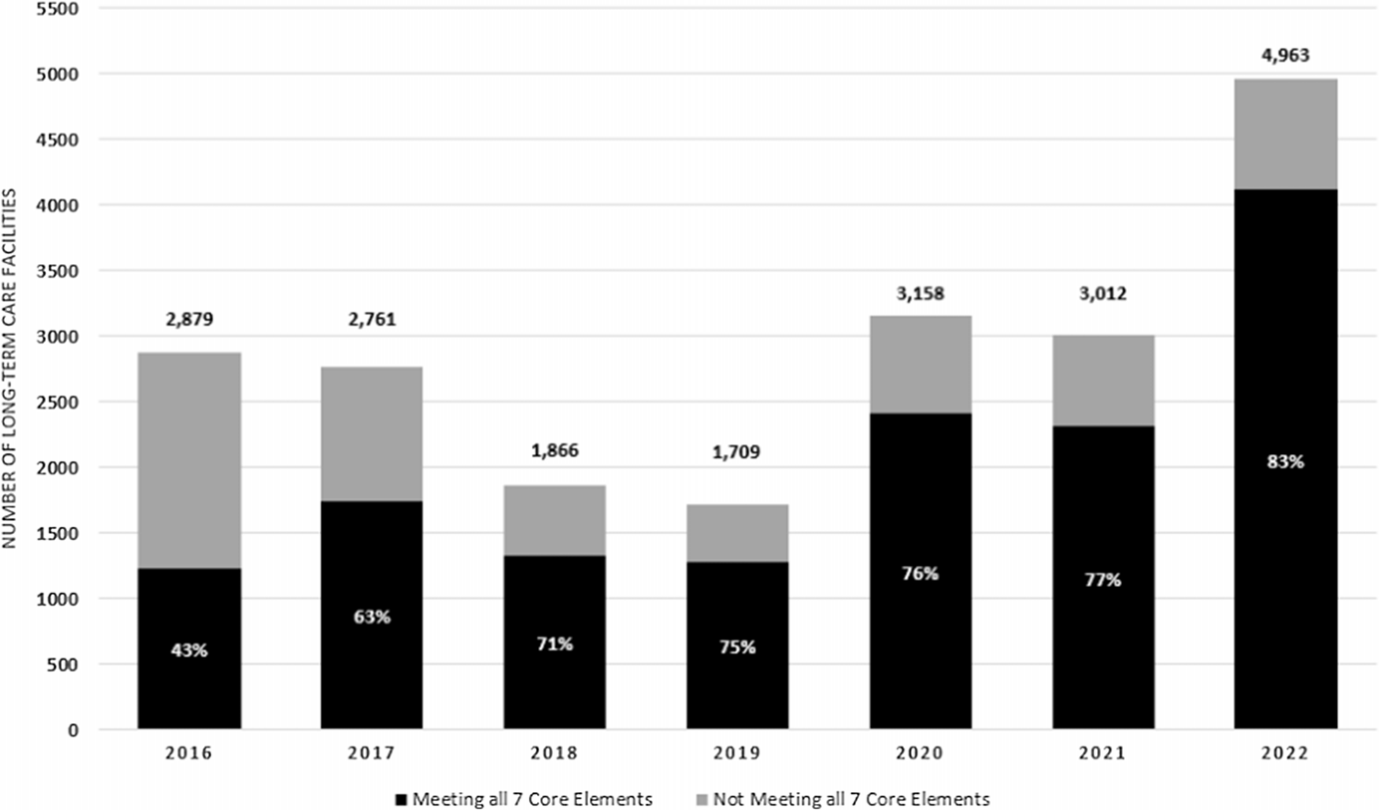
Reported Uptake of all Seven Core Elements of Antibiotic Stewardship in Long-term Care Facilities (LTCFs) Reporting to the National Healthcare Safety Network (NHSN), 2016–2022. Data Source: National Healthcare Safety Network Long-term Care Facility Annual Facility Surveys. Uptake of a Core Element was defined as affirmative response to at least one corresponding survey question. The percent on the bar graph indicates the percent of long-term care facilities implementing all 7 Core Elements each year.

A total of 12,842 surveys submitted to NHSN during 2019–2022 were included in the analysis (Appendix Figure 1). The number of facilities reporting in 2022 (n = 4,963) was almost 3 times the number of facilities in 2019 (n = 1,709). Of all reporting LTCFs in 2022, most had a bed size of 50–200 beds (80%), were in the Midwest (35%), chain-affiliated (48%), and had for-profit ownership (65%) (Appendix Table 1). The reported uptake of all seven Core Elements in LTCFs was 83% in 2022, an 8% increase from facilities reporting in 2019 (Table 1, Figure 1). There were 733 consecutive reporters from 2019 to 2022 (16% of facilities reporting in 2022), and the reported uptake of the seven Core Elements among consecutive reporters increased by 3% from 74% (Appendix Table 1). In 2022, the tracking, leadership, action, and accountability Core Elements all had greater than 98% uptake (Table 1). The most common roles responsible for stewardship activities were directors of nursing (86%), infection preventionists (83%), and medical directors (77%); 64% of LTCFs had a shared antibiotic stewardship responsibility of these roles (Appendix Table 1). Drug Expertise uptake was 97%, a 4% increase from facilities reporting in 2019. The Reporting Core Element, which entails facilities have a formal procedure for reviewing courses of antibiotic therapy and communicate with prescribers on selection, dosing, and duration, maintained the lowest uptake among all Core Elements in 2022 (90%).

**Table 1.** Reported uptake of CDC’s core elements of antibiotic stewardship in long-term care facilities (LTCFs) reporting to the National Healthcare Safety Network (NHSN), 2019–2022^a^

Variable	2019		2020		2021		2022		Absolute Difference, %^b^
	No.	%	No.	%	No.	%	No.	%	2019–2022
LTCFs Completing NHSN Annual Facility Survey	1,709		3,158		3,012		4,963		
Reported Uptake of All 7 Core Elements	1,275	74.6	2,407	76.2	2,306	76.6	4,112	82.9	+8.2
Antibiotic Stewardship Core Elements									
Leadership	1,694	99.1	3,121	98.8	2,984	99.1	4,925	99.2	+0.1
Accountability	1,685	98.6	3,118	98.7	2,961	98.3	4,875	98.2	−0.4
Drug Expertise	1,589	93.0	3,026	95.8	2,882	95.7	4,813	97.0	+4.0
Action	1,683	98.5	3,125	99.0	2,979	98.9	4,918	99.1	+0.6
Tracking	1,687	98.7	3,130	99.1	2,987	99.2	4,951	99.8	+1.0
Reporting	1,429	83.6	2,749	87.1	2,635	87.5	4,483	90.3	+6.7
Education	1,591	93.1	2,801	88.7	2,697	89.5	4,618	93.1	0.0

a These data were extracted from the 2019–2022 NHSN LTCF Component Annual Facility Surveys.

b Due to the rounding of the percentages to one decimal place, the absolute difference calculations may not equal the difference of the percentages shown above.

In 2022, most facilities reported having access to an EHR (98%, n = 4,873), with capabilities for medication orders (96%, n = 4,770), medication administration (95%, n = 4,735), and antimicrobial susceptibility results (81%, n = 4,015). Of the 4,898 (99%) respondents that indicated having a system for tracking AU, the most frequently used data sources and methods were manual tracking (48%, n = 2,340), EHR (31%, n = 1,514), and pharmacy services (25%, n = 1,214). Responses that indicated “other” (n = 271) included a combination of these three systems, as well as utilizing various software platforms for infection prevention and control. Of facilities with EHR medication order or administration capabilities, 28% (1,347/4,793) also reported using EHR for AU tracking.

## Conclusions

There was an 8% increase in the reported uptake of Core Elements of Antibiotic Stewardship in LTCFs from 2019 to 2022 and a 40% increase since reporting uptake of the Core Elements began in 2016. This may be due to regulatory antibiotic stewardship requirements and state-specific reporting requirements.^2^ State-level Core Element uptake data from 2016 to 2022 are publicly available on the CDC’s Antibiotic Resistance and Patient Safety Portal (https://arpsp.cdc.gov/profile/ltc/united-states-United%20States). Health departments and organizations can leverage these data to identify opportunities for supporting stewardship implementation in LTCFs.

The uptake of the Drug Expertise Core Element increased in 2022, and two-thirds of LTCFs reported having a consultant pharmacist responsible for improving the use of antimicrobials. Consultant pharmacists play a crucial role in supporting the implementation of stewardship activities by performing antimicrobial reviews and providing staff education and feedback on prescribing practices.^6–8^ Over 95% of LTCFs reported having EHR capabilities for tracking medication orders or administrations in 2022. However, less than one-third used their EHR for AU tracking, and nearly half reported manual tracking of AU. Increased EHR implementation has been shown to improve the quality of care in LTCFs and serves as a useful tool for clinicians.^9^ Electronic reporting of AU could reduce burden on LTCF staff and provide facilities with standardized AU reports to identify opportunities to optimize prescribing practices and evaluate the effectiveness of stewardship interventions.

This analysis has several limitations. Reporting to the NHSN LTCF Component Annual Facility Survey is voluntary; approximately one-third of US LTCFs submitted a survey. Therefore, these results may not be generalizable. Though we observed increased uptake of the Core Elements over the study period, nearly 2,000 new facilities started reporting in 2022, which may limit the ability to make comparisons over time. Due to CMS COVID-19 reporting requirements, the number of LTCFs reporting to NHSN increased starting in 2020.^10^ Based on a sensitivity analysis, LTCFs reporting continuously throughout the study period (2019–2022) had smaller increases (+3%) in reported uptake when compared to all reporting facilities (Appendix Table 2). Lastly, survey responses were not validated and may be influenced by social desirability bias leading to recording answers that may be more in-line with CMS requirements and CDC guidance than actual practices.

Uptake of the Core Elements of Antibiotic Stewardship in LTCFs increased from 2019 to 2022 and may indicate increased awareness of the importance of antibiotic stewardship for resident safety. Opportunities for integrating drug expertise in LTCFs automated tracking and reporting of AU may further support LTC staff in the implementation of stewardship activities to optimize the treatment of infection and resident care.

## Supplementary material

For supplementary material accompanying this paper visit http://doi.org/10.1017/ash.2025.31. click here to view supplementary material

## Supporting information

Luciano et al. supplementary material Luciano et al. supplementary material
