# Supplementary material for: Implementation of core elements of antibiotic stewardship in long-term care facilities—National Healthcare Safety Network, 2019–2022
Source: Antimicrob Steward Healthc Epidemiol. 2025 Mar 24;5(1):e86. doi: 10.1017/ash.2025.31 (PMC11951231; doi:10.1017/ash.2025.31)

# Appendix

| **Appendix Table 1. Characteristics of Long-term Care Facilities (LTCFs) Reporting to the National Healthcare Safety Network (NHSN) in 2022.*** | |
| --- | --- |
| **Characteristic** | **NHSN 2022** |
| Total No. of LTCFs reporting in 2022 | 4,963 |
| **Ownership, No. (%)** |  |
| For profit | 3,201 (64.5) |
| Not for profit, including government but not Veterans Affairs | 1,762 (35.5) |
| **Affiliation, No. (%)** |  |
| Multi-facility organization | 2,360 (47.6) |
| Independent | 2,126 (42.8) |
| Hospital system | 477 (9.6) |
| **No. of Beds, No. (%)** |  |
| <50 | 724 (14.6) |
| 50-99 | 1,930 (38.9) |
| 100-199 | 2,031 (40.9) |
| 200+ | 278 (5.6) |
| **US Census Region, No. (%)** |  |
| Midwest | 1,738 (35.0) |
| South | 1,574 (31.7) |
| Northeast | 961 (19.4) |
| West | 690 (13.9) |
| **EHR Access, No. (%)** | 4,873 (98.2) |
| **Total staff hours per week dedicated to infection prevention activity in the facility median (IQR)** | 30 (20-40) |
| **Data Source for Tracking Antimicrobial Use, No. (%)** |  |
| Manual tracking | 2,340 (47.8) |
| EHR | 1,514 (30.9) |
| Pharmacy data | 1,214 (24.8) |
| Other | 271 (5.5) |
| **Role Responsible for Antibiotic Stewardship, No. (%)** |  |
| Director of nursing | 4,274 (86.1) |
| Infection preventionist | 4,104 (82.7) |
| Medical director | 3,831 (77.2) |
| Consultant pharmacist | 3,313 (66.8) |
| **Note:** LTCF, long-term care facility; NHSN, National Healthcare Safety Network; IRQ, interquartile range; EHR, electronic health record  *These data were extracted from the 2022 NHSN LTCF Component Annual Facility Survey | |

| **Appendix Table 2. Reported Uptake of CDC’s Core Elements of Antibiotic Stewardship in Long-term Care Facilities (LTCFs) Reporting to the National Healthcare Safety Network (NHSN) among Consecutive Reporters^±^, 2019-2022.*** | | | | | | | | | |
| --- | --- | --- | --- | --- | --- | --- | --- | --- | --- |
| **Variable** | **2019** | | **2020** | | **2021** | | **2022** | | **Absolute Difference, %^**^** |
|  | **No.** | **%** | **No.** | **%** | **No.** | **%** | **No.** | **%** | **2019-2022** |
| LTCFs Completing NHSN Annual Facility Survey | 733 |  | 733 |  | 733 |  | 733 |  |  |
| Reported Uptake of All 7 Core Elements | 540 | 73.7 | 524 | 71.5 | 552 | 75.3 | 567 | 77.4 | +3.7 |
| **Individual Core Elements of Antibiotic Stewardship** | | | | | | | | | |
| Leadership | 727 | 99.2 | 723 | 98.6 | 729 | 99.5 | 731 | 99.7 | +0.5 |
| Accountability | 721 | 98.4 | 722 | 98.5 | 727 | 99.2 | 727 | 99.2 | +0.8 |
| Drug Expertise | 680 | 92.8 | 692 | 94.4 | 694 | 94.7 | 702 | 95.8 | +3.0 |
| Action | 717 | 97.8 | 720 | 98.2 | 722 | 98.5 | 725 | 98.9 | +1.1 |
| Tracking | 721 | 98.4 | 723 | 98.6 | 728 | 99.3 | 732 | 99.9 | +1.5 |
| Reporting | 606 | 82.7 | 608 | 83.0 | 633 | 86.4 | 629 | 85.8 | +3.1 |
| Education | 682 | 93.0 | 646 | 88.1 | 657 | 89.6 | 672 | 91.7 | -1.3 |
| **Note:** LTCF, long-term care facility; NHSN, National Healthcare Safety Network  **Note:** Uptake of a Core Element was defined as affirmative response to at least one corresponding annual facility survey question.  **^±^**Consecutive reporters: subset of LTCFs that submitted a survey in all four years of the study period (2019–2022)  *These data were extracted from the 2019-2022 NHSN LTCF Component Annual Facility Surveys.  **Due to the rounding of the percentages to one decimal place, the absolute difference calculations may not equal the difference of the percentages shown above. | | | | | | | | | |

**Appendix Figure 1. Inclusion of Surveys from Long-term Care Facilities Reporting to the National Healthcare Safety Network (NHSN) Long-term Care Facility Component Annual Facility Survey**


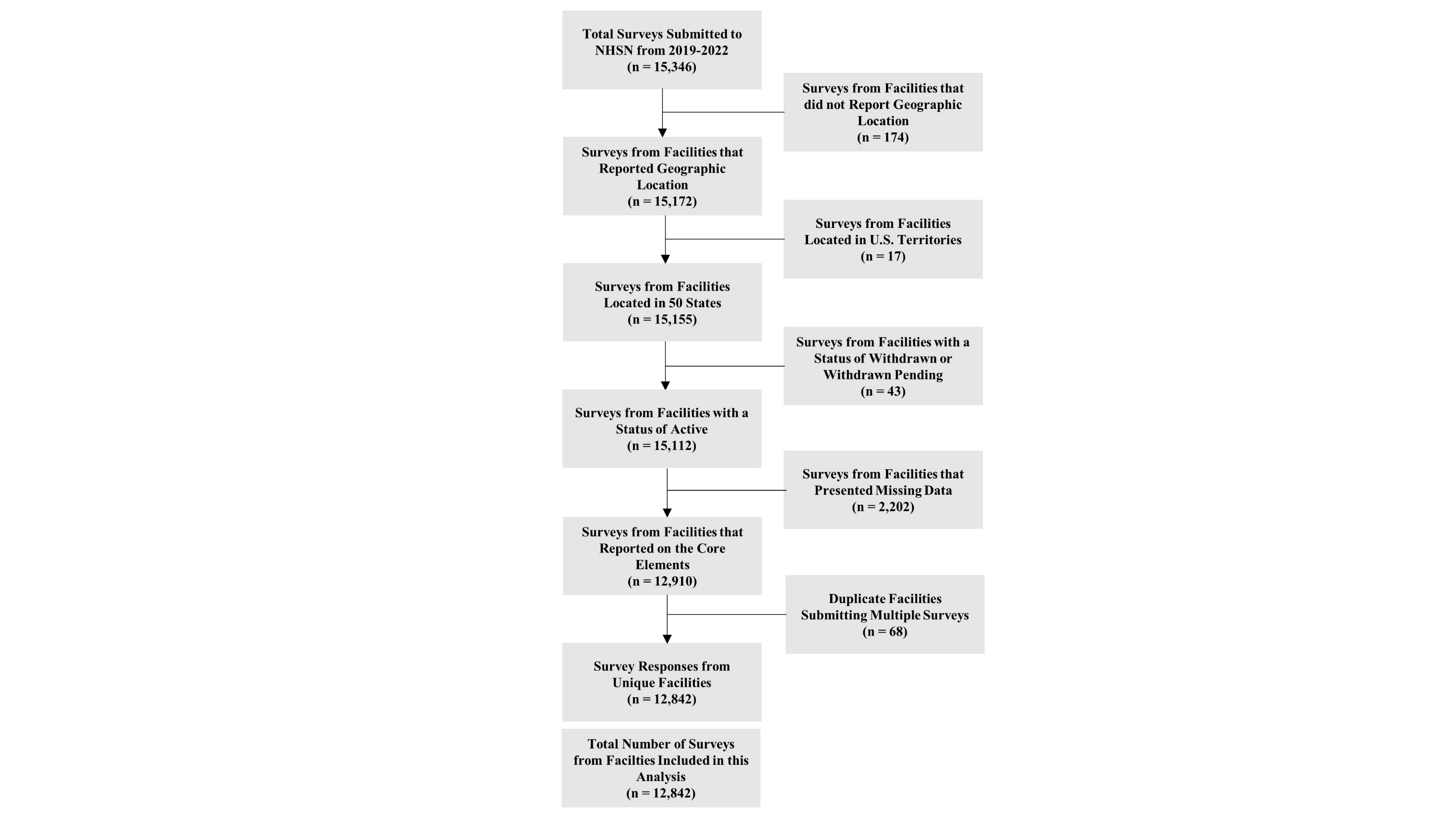

Supplement: Supplementary file 1 [file S2732494X25000312sup001.docx]
